# Dose and organ displacement comparisons with breast conservative radiotherapy using abdominal and thoracic deep‐inspiration breath‐holds: A comparative dosimetric study

**DOI:** 10.1002/acm2.13888

**Published:** 2023-01-07

**Authors:** Yoshitsugu Matsumoto, Etsuo Kunieda, Natsumi Futakami, Takeshi Akiba, Ryuta Nagao, Tsuyoshi Fukuzawa, Tomomi Katsumata, Toshihisa Kuroki, Tatsuya Mikami, Yoji Nakano, Yasuhiro Okumura, Kenji Souda, Eride Mutu, Akitomo Sugawara

**Affiliations:** ^1^ Department of Radiation Oncology Tokai University School of Medicine Isehara Kanagawa Japan; ^2^ Department of Radiation Oncology Tokai University Hachioji Hospital Hachioji Tokyo Japan; ^3^ Department of Radiology Tokai University Hospital Isehara Kanagawa Japan; ^4^ Course of Advanced Medical Science Graduate School of Medicine Tokai University Kanagawa Japan

**Keywords:** abdominal deep‐inspiration breath‐hold, body surface, breast cancer, radiation doses, radiotherapy, thoracic deep‐inspiration breath‐hold

## Abstract

Deep‐inspiration breath‐hold (DIBH) reduces the radiation dose to the heart and lungs during breast radiotherapy in cancer. However, there is not enough discussion about suitable breathing methods for DIBH. Therefore, we investigated the radiation doses and organ and body surface displacement in abdominal DIBH (A‐DIBH) and thoracic DIBH (T‐DIBH). Free‐breathing, A‐DIBH, and T‐DIBH computed tomography images of 100 patients were used. After contouring the targets, heart, and lungs, radiotherapy plans were created. We investigated the heart and lung doses, the associations between the heart and left lung displacements, and the thorax and abdominal surface displacements. No significant differences were observed in the target dose indices. However, the heart and lung doses were significantly lower in A‐DIBH than in T‐DIBH for all the indices; the mean heart and lung doses were 1.69 and 3.48 Gy, and 1.91 and 3.55 Gy in A‐DIBH and T‐DIBH, respectively. The inferior displacement of the heart and the left lung was more significant in A‐DIBH. Therefore, inferior expansion of the heart and lungs may be responsible for the respective dose reductions. The abdominal surface displaced more than the thoracic surface in both A‐DIBH and T‐DIBH, and thoracic surface displacement was greater in T‐DIBH than in A‐DIBH. Moreover, A‐DIBH can be identified because abdominal surface displacement was greater in A‐DIBH than in T‐DIBH. In conclusion, A‐DIBH and T‐DIBH could be distinguished by comparing the abdominal and thoracic surfaces of A‐DIBH and T‐DIBH, thereby ensuring the implementation of A‐DIBH and reducing the heart and lung doses.

## 1 INTRODUCTION

Radiotherapy is usually considered following breast‐conserving surgery, since it can reduce intra‐breast cancer recurrence and improve patient survival.^1^, ^2^ Randomized trials have found little difference in the local control and survival outcomes between patients treated with conventionally fractionated whole breast irradiation (WBI) and those receiving hypofractionated‐WBI,^3^ hypofractionated‐WBI has been widely used.^4^ Moreover, the non‐inferiority of partial‐breast and reduced‐dose radiotherapy compared with hypofractionated‐WBI was demonstrated in patients with early breast cancer.^5^ At the same time as the development of irradiation methods, the importance of reducing the heart dose has been emphasized, since recent research has shown that irradiation of the heart increases the risk of cardiac illness.^6^, ^7^ Therefore, intensity‐modulated radiotherapy (IMRT) and volumetric modulated arc therapy (VMAT) have been investigated to improve target dose conformity, and reductions in heart and lung doses.^8^, ^9^, ^10^, ^11^, ^12^

Meanwhile, the deep‐inspiration breath‐hold (DIBH) technique has been used as an alternative method to effectively reduce the heart and lung doses by enlarging the lungs and inherently increasing the gap between the heart and the irradiation field.^13^, ^14^, ^15^, ^16^, ^17^, ^18^, ^19^, ^20^ Previous reports on DIBH have focused on the effects of free‐breathing and deep inhalation; however, there are few reports on the abdominal and thorax deep‐inspiration breath‐hold (A‐DIBH and T‐DIBH) techniques. A‐DIBH is a breathing technique that uses the intercostal muscles to raise and lower the ribs, and T‐DIBH is a breathing technique consisting of raising and lowering the diaphragm. According to Zhao et al., abdominal breathing can reduce the heart dose more efficiently than thoracic breathing.^16^ Additionally, Hirata et al. evaluated doses to the heart, left lung, and descending coronary artery using A‐DIBH and T‐DIBH and found no significant differences between the two methods.^19^ However, both studies refer to the small number of people surveyed as a limitation (sample sizes of 25 and 14, respectively). Moreover, in A‐DIBH and T‐DIBH of left‐sided breast cancer, the correlation of organ displacements and body surface with heart and lung doses remains unclear.

Our institute performs DIBH for left breast cancer treatment using AlignRT (Vision RT, London, UK), which offers the accuracy of a commercially available optical three‐dimensional surface imaging system. Computed tomography (CT) images are normally taken using both A‐DIBH and T‐DIBH in addition to free‐breathing. Oncologists make the final decision as to which CT images to use for treatment.

In this study, replanning and dosimetry analysis were performed to examine the differences in the heart and lung doses using CT data from 100 patients performing A‐DIBH and T‐DIBH. In addition, using CT images, we analyzed the displacements of the heart, lungs, and body surface during A‐DIBH and T‐DIBH. We evaluated their correlation with heart and lung radiation doses.

## 2 MATERIALS AND METHODS

### 2.1 Patients and CT acquisition

A total of 120 patients who received radiotherapy with DIBH following breast‐conserving surgery for breast carcinoma of the left side between April 2018 and March 2020 were evaluated. Twenty patients were excluded because the patients themselves declared that A‐DIBH and T‐DIBH could not be performed properly. The mean age of patients was 57.8 years (28–78 years), and the median age was 58 years. Table 1 summarizes the demographic characteristics of the patient cohort. The institutional review board of our institution (20R089) approved this retrospective study protocol, and informed consent was waived owing to the retrospective nature of the study. The patients underwent CT (SOMATOM Definition AS, Siemens Healthcare, Forchheim, Germany) in the supine position with both arms raised using support tools. For CT simulation, metal markers were placed on the mid‐chest line, on the left mid‐axillary line, at the inferior border of the sternoclavicular joint, and 1 cm inferior to the inferior border of the breast. Before imaging, a radiation therapist trained the patient to breathe with their abdomen in A‐DIBH and to breathe with their thoracic in the T‐DIBH. Moreover, the radiation therapist instructed to inhale and stop whenever possible with A‐DIBH and T‐DIBH. The radiation therapist determined that a patient could perform both A‐DIBH and T‐DIBH if their thoracic and abdominal surface displacement differed between A‐DIBH and T‐DIBH. For each breathing technique, 3‐mm slices were obtained, first for free‐breathing without breath holding, and later for DIBH (A‐DIBH and T‐DIBH in no particular order).

**TABLE 1:** Characteristics of patients who received radiotherapy with A‐DIBH or T‐DIBH (*n* = 100)

		Median years (range)
Age		57.8 (28–78)
		Number of patients
Gender	Male	0
	Female	100
Histological diagnosis	Invasive ductal carcinoma	81
	Ductal carcinoma in situ	17
	Unknown	2
Stage	0	19
	I	50
	IIA	23
	IIB	7
	IIIA	1
	IIIB	0
	IIIC	0
	IV	0
	Not applicable	0
Breast dose/Fraction	40.05 Gy/15 fractions	80
	50 Gy/25 fractions	20
Boost dose/Fraction	10 Gy/4 fractions	70
	10 Gy/5 fractions	13
	None	17
DIBH respiratory method	A‐DIBH	48
	T‐DIBH	52
		Height and weight
Hight (cm)		157.3 ± 5.1
Weight (kg)		55.8 ± 9.5
BMI (kg/m2)		22.6 ± 3.8
Unknown		24

Abbreviations: A‐DIBH, abdominal deep‐inspiration breath‐hold; BMI, body mass index; T‐DIBH, thoracic deep‐inspiration breath‐hold.

### 2.2 Contouring and planning

For free‐breathing CT images, the whole heart (from the origin of the pulmonary artery to the apex) and the left lung were delineated, and for A‐DIBH and T‐DIBH CT images, the heart, the right and left lungs, the left anterior descending (LAD) and the left ventricle (LV) were delineated as organs‐at‐risk (OARs). The clinical target volume (CTV) was contoured by radiologists according to the ESTRO consensus guideline^20^ using A‐DIBH or T‐DIBH images (the CTV delineated on the A‐DIBH or T‐DIBH images used in the treatment). Furthermore, CTV was cropped within 5 mm of the skin contour. The CTV was transformed using MIM Maestro software (MIM Software, Cleveland, OH) onto other images (A‐DIBH to T‐DIBH or vice versa) to minimize the variations in delineation. The CTV was also cropped within 5 mm of the skin contour and subsequently confirmed by medical physicists. Then, the planning target volume (PTV) was created by expanding 5 mm from all sides of the CTV. The treatment plans for A‐DIBH and T‐DIBH were made using two tangential beams with a radiotherapy treatment planning system (RTPS; Eclipse version 13.7; Varian Medical Systems, Palo Alto, CA). A 6 MV X‐ray was generally used, and in cases when the maximum dose was too high, a 15 MV X‐ray was used in the field. The dose was calculated using the analytical anisotropic algorithm (AAA; Version 13.7). The gantry angle was set to coincide with the markers on the mid‐chest line and left axilla. For the superior edge of the radiation field, the inferior border of the humeral head was set, and for the inferior edge of the radiation field, the marker attached to the inferior side of the breast was set. If the PTV was superior to the humeral head, the radiation field was expanded until it reached the PTV. For the outside of the radiation field, the radiation field was opened by 1.5–2 cm from the body surface. Since this study was a dosimetric study, for the inside of the radiation field, the multi‐leaf collimator (MLC) was fitted according to the mid‐chest marker to prevent variation in MLC settings. Dose calculation was performed using the anisotropic analytical algorithm. The prescription dose to the PTV was 40.05 Gy delivered in 2.67 Gy per fraction,^21^, ^22^ and the plans were normalized to a dose of 98% volume (D98%) using the CTV, corresponding to a dose of 93% of the prescription dose. Some sub‐fields were created to prevent the administration of a high dose; the constraint was D2% of the PTV was <105% of the prescribed dose (40.05 Gy).

### 2.3 Analysis of dose indices

The dose indices of A‐DIBH and T‐DIBH were compared. The homogeneity indices (HI) were calculated by the ratio of the difference of the maximum (D2%) and minimum doses (D98%) to D50% (Equation 1). D98%, D95%, D50%, and D2% were compared for the PTV. The OAR endpoints were mean dose (D_mean_), volume, volumes receiving 30 Gy (V30 Gy), V20 Gy, V10 Gy, and V5 Gy for the heart and the lungs; maximum dose (D_max_), D_mean_, and V15 Gy for LAD and LV.

(1) $$HI\;=\;\frac{D2\%-D98\%}{D50\%}$$

### 2.4 Analysis of organ displacements

**FIGURE 1:**
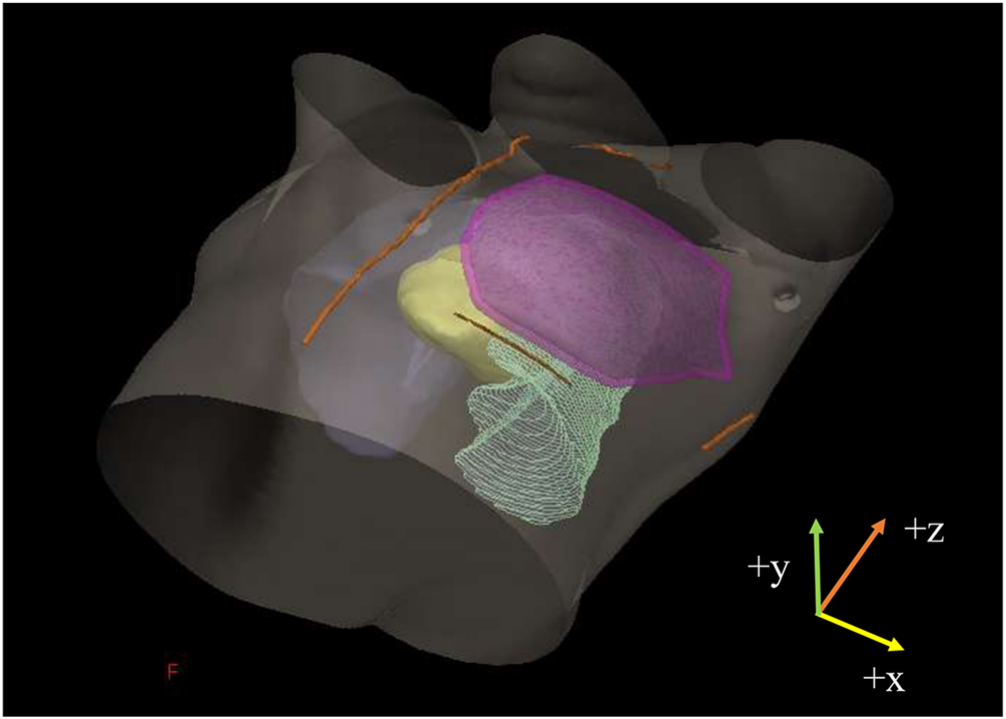
Three‐dimensional image of the thorax. Right and left lungs (gray and green, respectively), planning target volume (pink), surface markers (orange), and heart (yellow) are delineated. The positive axes of heart and lung displacements indicate the superior (z), anterior (y), and right (x) directions

The center coordinates of the heart, left lung (as the lung displacement), and PTV was retrieved using free‐breathing, A‐DIBH, and T‐DIBH CT images on the RTPS. The displacement of the center coordinates of the heart and left lung in the x, y, and z directions (right‐left, anterior‐posterior, and superior–inferior, respectively) during A‐DIBH and T‐DIBH was calculated from the difference of the center coordinates of heart and left lung between free‐breathing and each DIBH CT images (Figure 1). The differences in the shift of the central coordinates of the heart and left lung were compared during A‐DIBH and T‐DIBH. Moreover, the correlation between the displacement of the center coordinates of the heart and left lung and the radiation doses to the heart and lung was investigated. The distances between the center coordinates of the heart and PTV were calculated during A‐DIBH and T‐DIBH to compare the difference between the two methods in PTV‐heart distance due to displacement of the PTV and heart.

### 2.5 Analysis of body surface displacements

**FIGURE 2:**
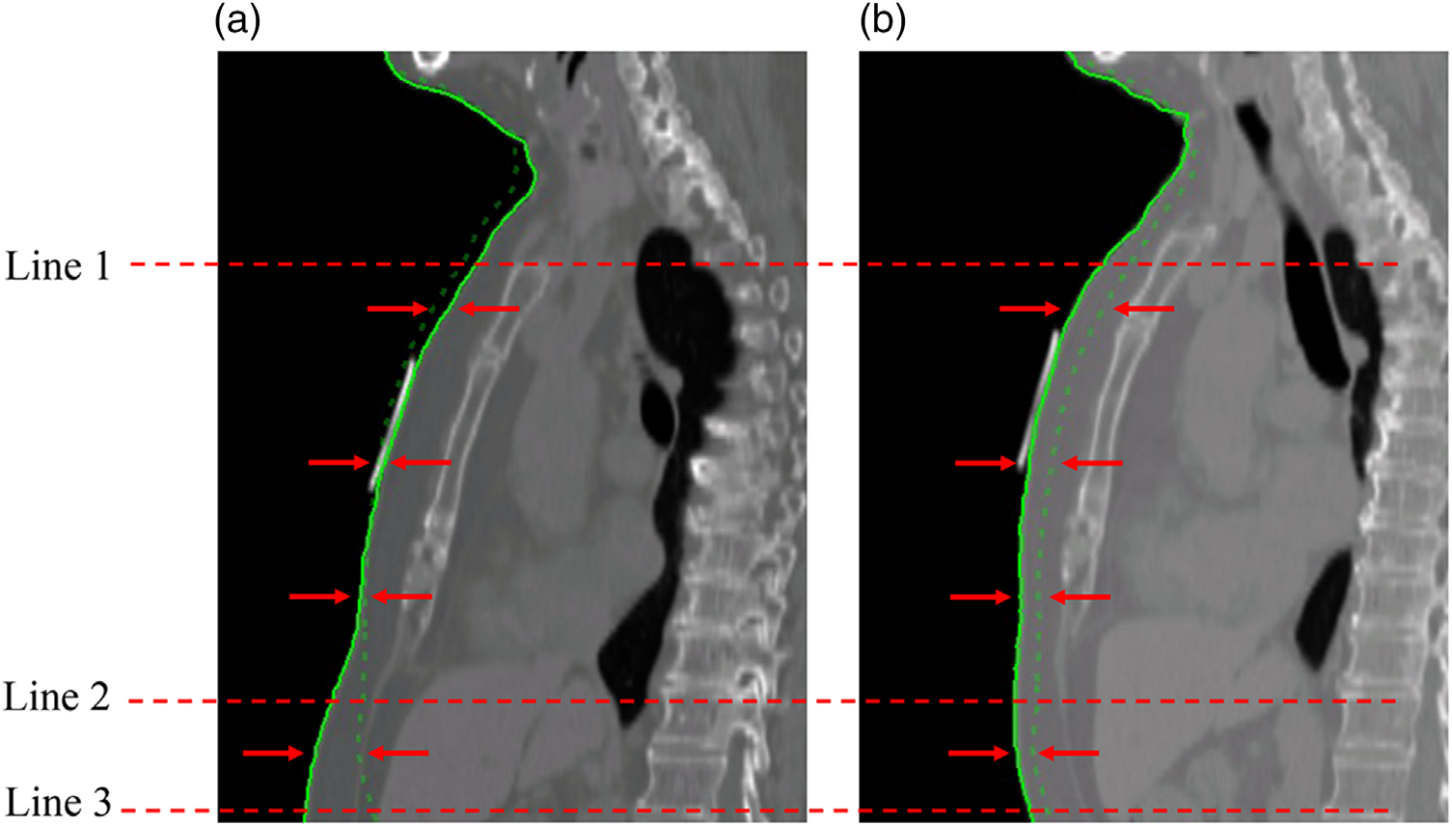
The grayscale image shows the A‐DIBH (a) and T‐DIBH (b) images. The dotted green lines represent the body contour during free‐breathing, and the solid green lines represent the body contour during DIBH. Line 1 shows the marker line at the inferior border of the sternoclavicular joint, and line 2 shows the marker line at 1 cm inferior to the inferior border of the breast. The abdomen was defined as 9 cm inferior to the thorax (line 3). The gap indicated by the red arrows is the length of the variation from free‐breathing to DIBH. A‐DIBH, abdominal deep‐inspiration breath‐hold; T‐DIBH, thoracic deep‐inspiration breath‐hold

The CT data and contour structures from free‐breathing, A‐DIBH, and T‐DIBH were transferred to matRad software (v2.10.1),^23^ an open‐source cross‐platform radiation treatment planning toolkit in MATLAB (The MathWorks, Natick, MA). A thorax‐to‐abdomen contour was extracted from the sagittal image plane based on the mid‐chest line marker during free‐breathing, A‐DIBH, and T‐DIBH. The thorax was defined by a marker placed below the sternoclavicular joint (Figure 2, line 1) to a marker 1 cm inferior to the lower breast (Figure 2, line 2), and the abdomen was defined as 9 cm inferior to the thorax (Figure 2, line 3) using the free‐breathing contour. The inferior coordinates of line 2 were matched on the contours of free‐breathing, A‐DIBH, and T‐DIBH. The differences between the body surface displacements from free‐breathing to A‐DIBH and T‐DIBH in the direction perpendicular to the body axis at 1.5 mm intervals were calculated and obtained average and maximum values. Correlations were investigated between the heart and lung mean doses and the displacement of the thorax and abdominal surfaces in A‐DIBH and T‐DIBH. Moreover, differences in the displacement of the thorax and abdominal surfaces between A‐DIBH and T‐DIBH were investigated.

### 2.6 Effect of the cardiac shielding by MLC

In clinical practice, the MLC is placed beyond the midline to reduce heart doses as required. Therefore, a subset analysis was performed using 20 consecutive patients. Plans were created in which the heart were shielded with MLC as much as possible without shielding the PTV with A‐DIBH, and the doses were compared between the original A‐DIBH plans and the plans in which the heart was shielded with MLC with T‐DIBH. All plans were normalized to a dose of 98% volume (D98%) using the CTV, corresponding to a dose of 93% of the prescription dose.

### 2.7 Effect of the calculation algorithm

To investigate the effect of the calculation algorithm on the dose, the calculation algorithm was changed to Acuros XB (AXB; Version 13.7) and recalculated in a subset of 20 consecutive patients. Plans were calculated using the AXB algorithm with A‐DIBH, and the doses were compared between the original A‐DIBH plans, which were calculated using AAA and the T‐DIBH plans, which were calculated using the AXB algorithm. In all plans, the monitor units were set to the same value for both AAA and AXB algorithms.

### 2.8 Statistical analysis

The two‐sided Wilcoxon signed‐rank test was used to determine the differences in the dose indices of the target and OARs for both A‐DIBH and T‐DIBH and in dose comparisons of the subset analyses. It was also used to determine the differences in the shift of the central coordinates of the heart, left lung, and the differences in PTV ‐ heart distance to A‐DIBH and T‐DIBH. Similarly, the differences between A‐DIBH and T‐DIBH with respect to the displacement of the thoracic and abdominal surfaces were evaluated. Additionally, Spearman's test was used to identify the correlation between the mean doses to the heart and lungs with the displacement of the heart, left lung, thorax, and abdominal surface. Statistical analysis was performed using IBM SPSS Statistics version 26.0 (IBM Corp., Armonk, NY). Statistical significance was set at *p* < 0.05.

## 3 RESULTS

There was no significant difference in PTV volume, Hounsfield unit, D98%, D95%, D50%, D2%, and HI between A‐DIBH and T‐DIBH (Table 2). For both the heart and lungs, the volume was significantly lower in A‐DIBH than in T‐DIBH, and dose indices were significantly lower in A‐DIBH for all indices of the mean dose, V30 Gy, V20 Gy, V10 Gy, and V5 Gy. The mean heart and lung doses were lower for A‐DIBH than for T‐DIBH in 72 and 74 of the 100 patients, respectively. In the other OAR indices, A‐DIBH was lower than T‐DIBH in approximately 70% of the patients (Table 2). The mean doses to the heart were 1.69 Gy and 1.91 Gy in A‐DIBH and T‐DIBH, and those to the lungs were 3.48 Gy and 3.55 Gy in A‐DIBH and T‐DIBH, respectively.

**TABLE 2:** Comparison between A‐DIBH and T‐DIBH for each index

Parameters		A‐DIBH	T‐DIBH	*p*‐value
PTV				
	Volume (cm^3^)	487.05 (110.00–1128.35) {50}	493.89 (104.91–1112.63) {50}	0.538
	Hounsfield unit	–70.05 (–114.74 to 8.19) {53}	–70.67 (–112.56 to 8.46) {47}	0.071
	D98% (Gy)	22.82 (16.31–36.46) {54}	22.27 (14.82–36.14) {65}	0.086
	D95% (Gy)	29.89 (22.92–36.90) {55}	29.31 (22.20–36.80) {45}	0.065
	D50% (Gy)	39.38 (37.96–40.22) {46}	39.38 (38.07–40.53) {54}	0.052
	D2% (Gy)	41.43 (39.95–41.94) {51}	41.43 (39.63–41.87) {48}	0.664
	HI	0.47 (0.13–0.63) {47}	0.49 (0.14–0.66) {53}	0.100
Heart				
	Volume (cm^3^)	417.10 (264.40–629.50) {20}	440.45 (300.40–688.80) {80}	**0.000** **
	Mean (Gy)	1.69 (0.56–9.03) {28}	1.91 (0.56–8.77) {72}	**0.000** **
	V30 Gy (%)	0.91 (0.00–18.25) {22}	1.42 (0.00–17.38) {62}	**0.000** **
	V20 Gy (%)	1.65 (0.00–21.36) {27}	2.25 (0.00–20.55) {73}	**0.000** **
	V10 Gy (%)	2.60 (0.00–24.38) {25}	3.37 (0.00–23.81) {70}	**0.000** **
	V5 Gy (%)	4.31 (0.00–28.11) {25}	4.98 (0.00–27.01) {75}	**0.000** **
Lungs				
	Volume (cm^3^)	3880.80 (1986.00–5892.20) {35}	4075.20 (2733.70–5807.00) {65}	**0.007** **
	Mean (Gy)	3.48 (1.19–5.76) {26}	3.55 (1.23–6.14) {74}	**0.000** **
	V30 Gy (%)	5.76 (0.90–11.60) {24}	6.06 (0.00–18.25) {76}	**0.000** **
	V20 Gy (%)	7.60 (1.49–13.86) {24}	8.02 (1.49–13.86) {76}	**0.000** **
	V10 Gy (%)	9.40 (2.37–15.85) {25}	9.62 (2.37–15.85) {75}	**0.000** **
	V5 Gy (%)	12.43 (4.50–21.25) {27}	13.20 (4.57–21.07) {73}	**0.000** **
LAD	Maximum (Gy)	36.88 (3.12–40.61) {34}	37.83 (3.65–37.34) {66}	**0.000** **
	Mean (Gy)	12.84 (1.63–35.71) {30}	16.62 (1.66–37.79) {70}	**0.000** **
	V15 Gy (%)	35.73 (0.00–97.80) {29}	46.15 (0.00–100.00) {62}	**0.000** **
LV	Maximum (Gy)	37.63 (3.70–40.03) {44}	37.83 (3.65–40.99) {56}	**0.030** **
	Mean (Gy)	3.10 (0.85–17.30) {28}	3.73 (0.88–18.64) {72}	**0.000** **
	V15 Gy (%)	5.24 (0.00–45.77) {25}	7.34 (0.00–49.18) {65}	**0.000** **

*Note*: Data are presented as median (range). {} is the number of high indexes when comparing the two and excludes ties.

Abbreviations: A‐DIBH, abdominal deep‐inspiration breath‐hold; HI, homogeneity index; LAD, left anterior descending; LV, left ventricle; PTV, planning target volume; T‐DIBH, thoracic deep‐inspiration breath‐hold.

^*^ *p* < 0.05; **p < 0.01.

**FIGURE 3:**
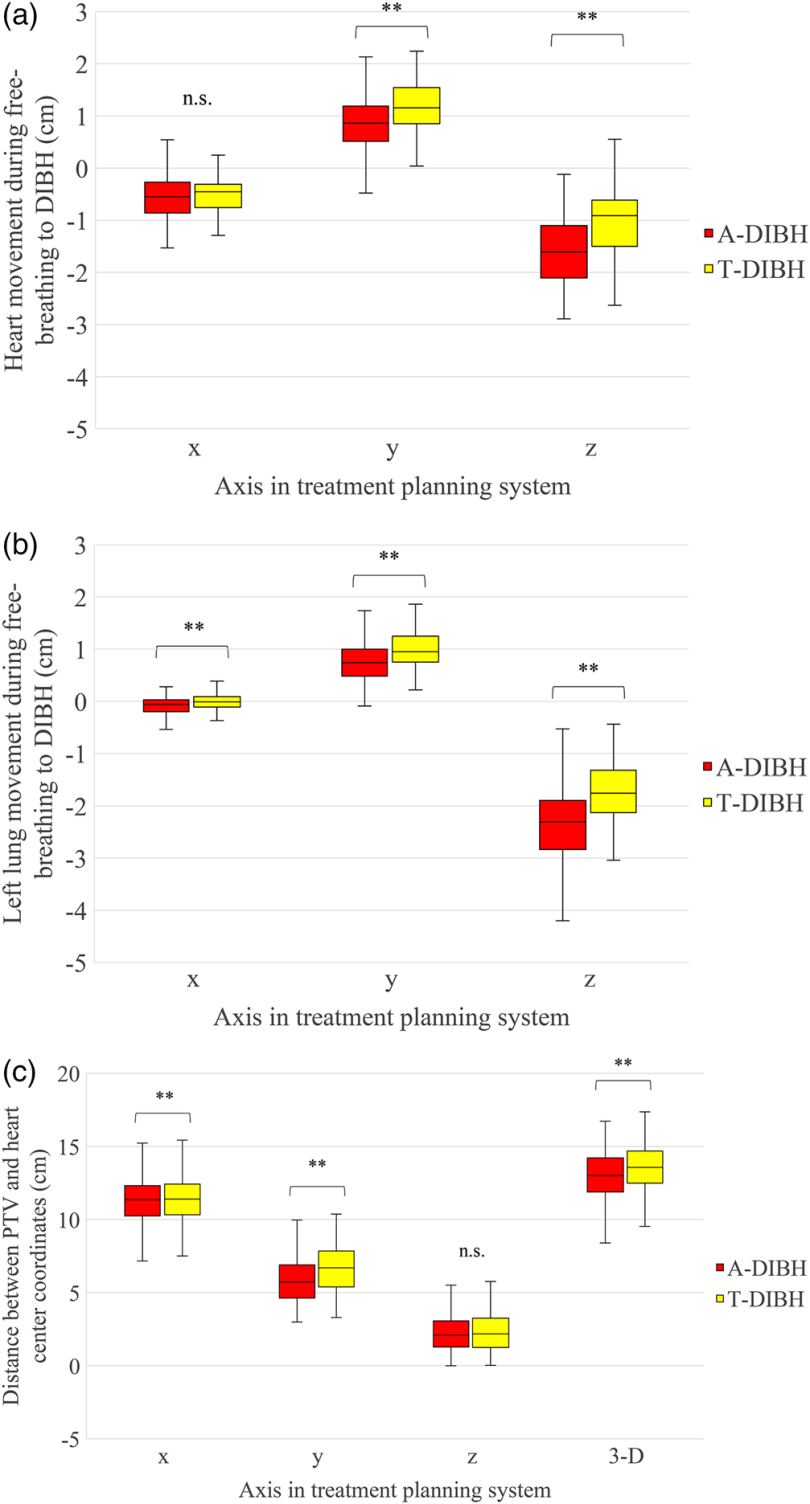
The difference in displacement of the heart (a) and left lung (b) for A‐DIBH and T‐DIBH. The x‐axis shows the displacement in the right‐to‐left direction (+x pointed left), the y‐axis shows displacement in the anterior‐to‐posterior direction (+y pointed anterior), and the z‐axis shows displacement in the superior‐to‐inferior direction (+z pointed superior). (c) The difference in the central coordinate of the heart and planning target volume for A‐DIBH and T‐DIBH. 3‐D, distance between two points in three dimensions. n. s, not significant. **p < 0.01

**FIGURE 4:**
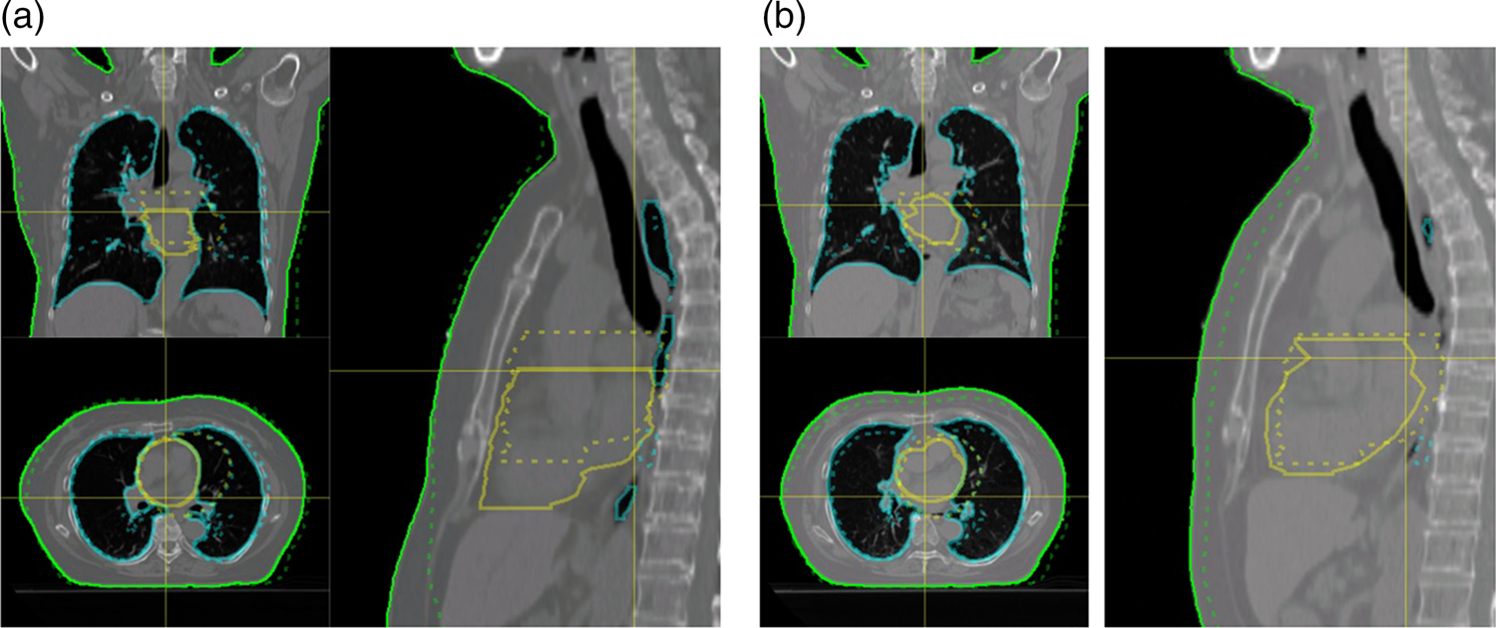
The fused images of A‐DIBH and the contour of free‐breathing image (a), T‐DIBH and the contour of free‐breathing image (b), respectively. The grayscale images show the DIBH images. The dotted lines represent the contours of organs during free‐breathing, and the solid lines represent the contours of organs during DIBH. Green indicates the body contour, cyan indicates the lung, and yellow is the heart. Coronal (upper left), axial (lower left), and sagittal (right) views are shown. A‐DIBH, abdominal deep‐inspiration breath‐hold; T‐DIBH, thoracic deep‐inspiration breath‐hold

As for organ displacement during both A‐DIBH and T‐DIBH, the heart and left lung were displaced toward the right, anterior, and inferior directions in many cases during inspiration. Specifically, the heart was displaced significantly lesser towards the anterior direction during A‐DIBH than during T‐DIBH, and more towards the inferior directions during A‐DIBH than during T‐DIBH. The left lung was also displaced significantly more towards the anterior direction during T‐DIBH and more towards the right and inferior direction during A‐DIBH. In the distances between the heart to PTV, there was no significant difference between A‐DIBH and T‐DIBH in the z‐axis. However, A‐DIBH was significantly shorter between A‐DIBH and T‐DIBH in the x‐ and y‐axes and distances in the three dimensions (Figures 3 and 4).

The mean heart dose was slightly correlated with the heart displacement in the anterior‐posterior and superior–inferior directions during A‐DIBH (y: *r* = −0.24, z: *r* = 0.21) and in the anterior‐ posterior direction during T‐DIBH (*r* = −0.21) (Table 3, Figure S1a). Furthermore, the left lung was displaced anteriorly and inferiorly during inspiration in most cases. The mean lung dose was slightly correlated with left lung displacement in the anterior‐posterior and superior‐inferior directions during both A‐DIBH (y: *r* = −0.36, z: *r* = 0.30) and T‐DIBH (y: *r* = −0.28, z: *r* = 0.26) (Table 3, Figure S1b).

**TABLE 3:** Correlation of heart and lung D_mean_ and heart and left lung central coordinate displacements

		x	y	z
		Axis of heart displacement		
Heart D_mean_ (A‐DIBH)	Correlation coefficient	0.14	**−0.24** *	**0.21** *
	*p*‐value	0.182	0.017	0.032
Heart D_mean_ (T‐DIBH)	Correlation coefficient	−0.02	**−0.21** *	0.13
	*p*‐value	0.854	0.034	0.215
		Axis of left lung displacement		
Lung D_mean_ (A‐DIBH)	Correlation coefficient	0.08	**−0.36** **	**0.30** **
	*p*‐value	0.461	0.000	0.002
Lung D_mean_ (T‐DIBH)	Correlation coefficient	−0.02	**−0.28** **	**0.26** **
	*p*‐value	0.837	0.004	0.009

Abbreviations: 3‐D, three‐dimensional; A‐DIBH, abdominal deep‐inspiration breath‐hold; T‐DIBH, thoracic deep‐inspiration breath‐hold.

^*^ *p* < 0.05; **p < 0.01.

**FIGURE 5:**
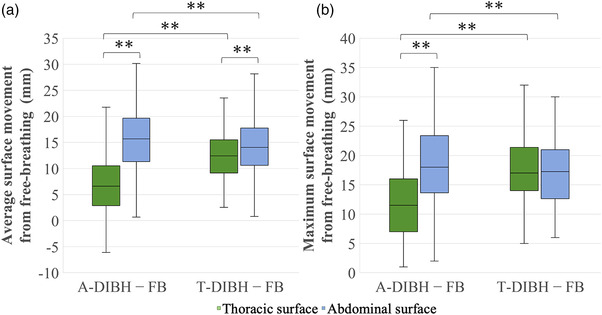
The average thoracic and abdominal surface displacements from free‐breathing to A‐DIBH and T‐DIBH (a). The maximum thoracic and abdominal surface displacements from free‐breathing to A‐DIBH and T‐DIBH (b). A‐DIBH, abdominal deep‐inspiration breath‐hold; T‐DIBH, thoracic deep‐inspiration breath‐hold; FB, free‐breathing. **p < 0.01

There was a slightly negative correlation between the heart and lung mean doses and the average abdominal surface displacement for both A‐DIBH and T‐DIBH (heart: *r* = −0.34, *r* = −0.35, respectively; lung: *r* = −0.19, *r* = −0.24, respectively). Moreover, there was a slightly negative correlation between the heart mean doses and the maximum abdominal surface displacement for both A‐DIBH and T‐DIBH (heart: *r* = −0.30, *r* = −0.26, respectively). However, no correlation was observed between the heart or lung mean doses and the average and maximum thoracic surface displacement in both conditions (Table 4, Figure S2a,b). The average thoracic and abdominal surface median displacements were 6.61 ± 5.69 (−6.09 to 28.36) mm and 15.68 ± 6.77 (−1.71 to 43.30) mm in A‐DIBH, respectively, and 12.42 ± 4.91 (2.57 – 30.98) mm and 14.07 ± 5.73 (0.81 – 39.21) mm in T‐DIBH, respectively. Average abdominal surface displacements were significantly larger than the average thoracic surface displacements in both A‐DIBH and T‐DIBH and the number of the patients in whom the average abdominal displacement was greater than the average thoracic displacement was 96 and 64 patients in A‐DIBH and T‐DIBH, respectively. None of the patients had the same abdominal and thoracic displacement in either group. Moreover, the medians of the displacement of A‐DIBH minus the displacement of T‐DIBH on the average thoracic and abdominal surfaces were −5.17 ± 5.9 (−22.27 to 4.77) mm and 1.2 ± 5.39 (−14.32 to 19.48) mm, respectively; there were significant differences in both the A‐DIBH and T‐DIBH average thoracic and abdominal surface displacements (Figure 5a). The maximum thoracic and abdominal surface displacements in A‐DIBH and T‐DIBH showed a similar trend. However, there were no significant maximum thoracic and abdominal surface displacements in T‐DIBH. (Figure 5b).

**TABLE 4:** Correlation of heart and lung D_mean_ and abdominal and thoracic surface displacements (Average and maximum)

		Abdominal surface displacement (Average)	Thoracic surface displacement (Average)
Heart D_mean_ (A‐DIBH)	Correlation coefficient	**−0.34** **	−0.10
	*p*‐value	0.001	0.345
Heart D_mean_ (T‐DIBH)	Correlation coefficient	**−0.35** **	−0.17
	*p*‐value	0.000	0.085
Lung D_mean_ (A‐DIBH)	Correlation coefficient	−0.19	−0.06
	*p*‐value	0.061	0.558
Lung D_mean_ (T‐DIBH)	Correlation coefficient	**−0.24** *	0.03
	*p*‐value	0.016	0.752

		Abdominal surface displacement (Maximum)	Thoracic surface displacement (Maximum)
Heart D_mean_ (A‐DIBH)	Correlation coefficient	**−0.30** **	−0.04
	*p*‐value	0.003	0.702
Heart D_mean_ (T‐DIBH)	Correlation coefficient	**−0.26** **	−0.09
	*p*‐value	0.010	0.373
Lung D_mean_ (A‐DIBH)	Correlation coefficient	−0.13	−0.08
	*p*‐value	0.212	0.427
Lung D_mean_ (T‐DIBH)	Correlation coefficient	−0.15	–0.13
	*p*‐value	0.129	0.186

Abbreviations: A‐DIBH, abdominal deep‐inspiration breath‐hold; T‐DIBH, thoracic deep‐inspiration breath‐hold.

^*^ *p* < 0.05, **p < 0.01.

Plans with heart shielding with MLC showed a dose reduction in all OARs, particularly in heart dose, from a median of 1.78 Gy to 1.10 Gy; A‐DIBH and T‐DIBH plan with heart shielding with MLC lost the advantage in all dose indices (Table S1). Due to differences in calculation algorithms, significant dose differences were found between AAA and AXB algorithms in several dose indices for PTV and OARs. Comparing A‐DIBH and T‐DIBH dose indices using the AXB algorithm showed no significant differences in lungs and LADs compared to the original comparison using 100 patients with AAA (Table S2).

## 4 DISCUSSION

There are few reports on whether A‐DIBH or T‐DIBH reduces heart and lung doses in the literature. Therefore, we performed a dosimetric comparison using CT images of DIBH radiotherapy in left‐sided breast cancer. We found no differences in the PTV dose indices between A‐DIBH and T‐DIBH. However, the heart and lung doses of A‐DIBH were significantly lower in all the indices, including D_mean_, V30 Gy, V20 Gy, V10 Gy, and V5 Gy. Moreover, LAD and LV doses of A‐DIBH were significantly lower in D_max_, D_mean_, and V15 Gy. Therefore, when treatment plans were similar and there was no significant difference in the PTV dose indices, A‐DIBH was associated with lower heart and lung doses than T‐DIBH. Hirata et al.^19^ found no significant differences between A‐DIBH and T‐DIBH in the heart and left lung doses in 14 patients with left breast cancer who underwent WBI after breast‐conserving surgery; the median heart doses using A‐DIBH and T‐DIBH were 1.6 Gy and 1.5 Gy for the entire treatment period, respectively, in a prescription dose of 50 Gy. Meanwhile, Zhao et al.^16^ found significant differences between A‐DIBH and T‐DIBH in the heart and left lung doses in 22 patients; the median heart doses using A‐DIBH and T‐DIBH were 1.34 Gy and 1.67 Gy, respectively, in a prescription dose of 50 Gy. The result of the present study is similar to that of Zhao et al.,^16^ in that the mean heart and lung doses were lower in A‐DIBH than in T‐DIBH. However, the heart doses observed in our study were higher than those in previous studies.^16^, ^19^ This may be because, in clinical practice, the MLC is placed beyond the midline to reduce the heart and lungs doses, if necessary. However, we adopted the basic method of aligning the irradiation field to the mid‐chest line without manually shielding the heart to reduce planning variation in this dosimetric study. The significant dose reduction in hearts in the subset analysis of plans that shielded the hearts with MLC, and the fact that the heart mean dose was close to them suggests that the hearts were shielded in the their reports.

The displacement of the central coordinates of the heart and left lung indicates the organ movement and direction of their expansion. The heart was displaced more toward the posterior and inferior directions in A‐DIBH than in T‐DIBH. Considering the relationship between the heart dose, displacement, the distance between the PTV and heart, although all the indices of the heart dose in A‐DIBH were low, the distance between the PTV and heart in A‐DIBH was shorter in the T‐DIBH on the x‐ and y‐axes, and the three‐dimensional distance and heart volume were low. Moreover, the inferior movement of the heart was more significant in A‐DIBH; it slightly correlated with a decrease in the mean dose to the heart. Therefore, there is a possibility that the heart was out of the irradiation field owing to inferior expansion and the changes in its shape regardless of the distance from the PTV in A‐DIBH. The whole heart is subjected to cardiac side effects, including LAD and the (LV),^24^ which have also been investigated in relation to DIBH.^25^, ^26^ In this study, not only the whole heart but also LAD and LV of all indices of the heart dose were lower in A‐DIBH, it is clear that the entire heart, including the surface of the heart close to the irradiation field, is out of the irradiation field. The patterns of lung displacement in the present study were similar to those observed in a previous study.^19^ The left lung was displaced more towards the anterior and inferior directions. The left lung was displaced more inferiorly in A‐DIBH compared to T‐DIBH and was displaced anteriorly in T‐DIBH compared to A‐DIBH. According to the result of the lung mean dose and displacement, since displacement in the inferior and anterior left lung correlates with a decrease in the lung dose, the inferior displacement of the left lung was considered the cause of the reduction in the lung dose at A‐DIBH. Since the lung volume of A‐DIBH was smaller than that of T‐DIBH, the inferior expansion of the left lung may have a more significant effect on the lung dose than the decrease in lung dose owing to the increase in the lung volume in A‐DIBH. Zhao et al. and Vikström et al. demonstrated that DIBH decreases the heart volume because the lung expansion in DIBH applies pressure to the heart.^16^, ^27^ Moreover, Zhao et al.^16^ reported that increased intrathoracic pressure could possibly reduce the heart dose. In the present study, the lung volume in T‐DIBH increased more than that in A‐DIBH. However, the heart dose in A‐DIBH decreased more than that in T‐DIBH, revealing that apart from the reduction in cardiac dose due to lung expansion, the effect of cardiac dose due to heart displacement and the changes in its shape may also be significant.

The correlation between the thoracic and abdominal surface displacements and the mean heart and lung doses suggested that abdominal surface displacement was an indicator of the degree of dose reduction to the heart and lungs, while this was not true of the thoracic surface displacements, which showed no correlation. Therefore, we believe it is possible to predict the heart and lung dose reduction effects from the magnitude of the abdominal displacement during DIBH. We found that the displacement of the abdominal surface was more significant than the thoracic surface in DIBH, regardless of whether the patients were using A‐DIBH or T‐DIBH. Moreover, abdominal surface displacement was greater in A‐DIBH than T‐DIBH, and thoracic surface displacement was greater in T‐DIBH than A‐DIBH. Therefore, A‐DIBH can be distinguished by comparing the abdominal and thoracic surfaces of both A‐DIBH and T‐DIBH. Oechsner et al. found that the body surface expansion toward the anterior direction correlated with the lung dose reduction,^14^ which was in line with our results. However, we found that the lung dose was correlated with the abdominal surface expansion toward the anterior direction, not with the thoracic surface. Zhao et al.^16^ argued that 72.7% of patients using DIBH defaulted to T‐DIBH when the breathing method was not specified. Therefore, A‐DIBH could be distinguished by observing the thoracic and abdominal surface displacements and performing A‐DIBH, a reduction in the heart and lung doses can be achieved.

The AXB algorithm is more accurate for calculating heterogeneous such as lungs surrounded by chest than the AAA.^28^ Some dose indices from subset analysis using AXB algorithms no longer show a significant difference in lung dose for A‐DIBH and T‐DIBH, suggesting that the actual difference between A‐DIBH and T‐DIBH lung dose difference is less than the dose calculated with AAA. The results of a subset analysis measured with the heart shielded by MLC. If cardiac shielding does not affect the target dose, in both A‐DIBH and T‐DIBH, the inferior heart and lungs are shielded by the MLC, and therefore there may be no significant difference in OAR doses for the target, heart, and lungs in A‐DIBH and T‐DIBH.

There are some limitations to this study. First, it is up to the therapists to decide whether patients can perform the A‐DIBH or T‐DIBH, and it is difficult hard to make that decision. Therefore, there is a possibility that some of the patients could not adequately perform A‐DIBH or T‐DIBH. However, the effects of this limitation were reduced by the large sample size of 100 patients. Second, A‐DIBH or T‐DIBH imaging was performed after imaging free‐breathing CT, and the sequence of A‐DIBH or T‐DIBH was in no particular order. Therefore, we could not consider the effect of the order of A‐DIBH and T‐DIBH on the heart and lung doses. Third, in clinical practice, T‐DIBH was chosen by nearly half of the patients. It shows that it is difficult to select from CT images better breathing techniques that result in lower heart and lung doses. Fourth, this study was conducted when the MLC did not shield the heart and set in the mid‐chest line. If the target dose is not affected, it may be possible to shield the heart with the MLC and reduce the heart and lung doses similarly for both A‐DIBH and T‐DIBH.

## 5 CONCLUSION

The results of our study demonstrated that A‐DIBH is associated with lower heart and lung doses than T‐DIBH, which may be due to the inferior expansion of the heart and lungs. Moreover, thoracic and abdominal surface displacements help distinguish between A‐DIBH and T‐DIBH. The abdominal surface displacement was greater than the thoracic surface displacement in both A‐DIBH and T‐DIBH; however, the thoracic surface displacement was smaller in A‐DIBH than in T‐DIBH. A‐DIBH should be implemented and radiation doses to the heart and lungs should be reduced.

## AUTHOR CONTRIBUTION

All co‐authors have reviewed and edited to the work and have given approval to the submission.

## CONFLICT OF INTEREST

No conflicts of interest.

## Supporting information

Supporting Material

Supporting Material

Supporting Material

Supporting Material

Supporting Material

Supporting Material

Supporting Material

Supporting Material

Supporting Material

## Data Availability

Data available on request due to ethical restrictions.
