# Supplementary figures and images for: Dose and organ displacement comparisons with breast conservative radiotherapy using abdominal and thoracic deep‐inspiration breath‐holds: A comparative dosimetric study
Source: J Appl Clin Med Phys. 2023 Jan 7;24(4):e13888. doi: 10.1002/acm2.13888 (PMC10113706; doi:10.1002/acm2.13888)

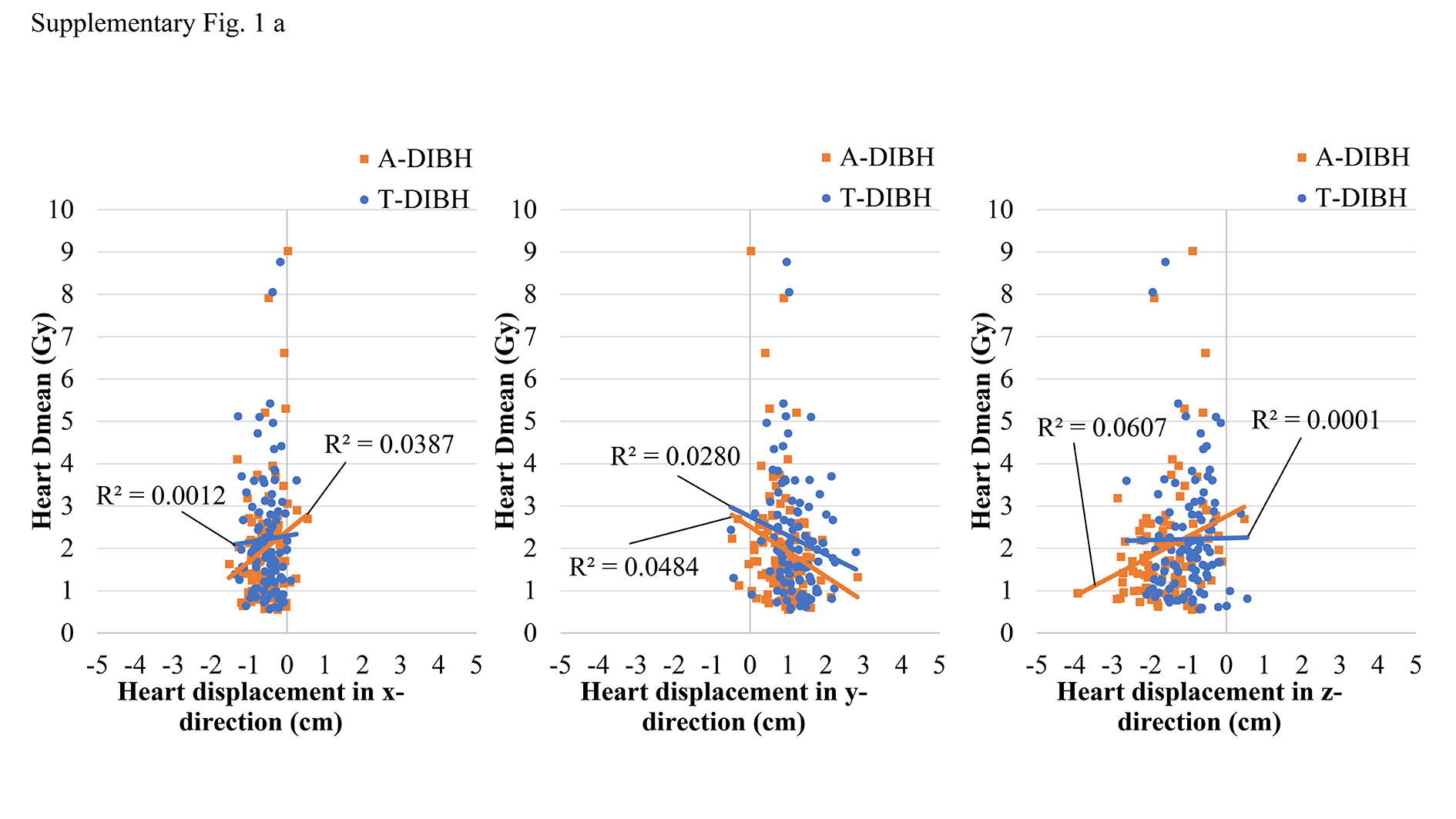

Supplement: Supplementary file 1 — Supporting Material [file ACM2-24-e13888-s009.tif]

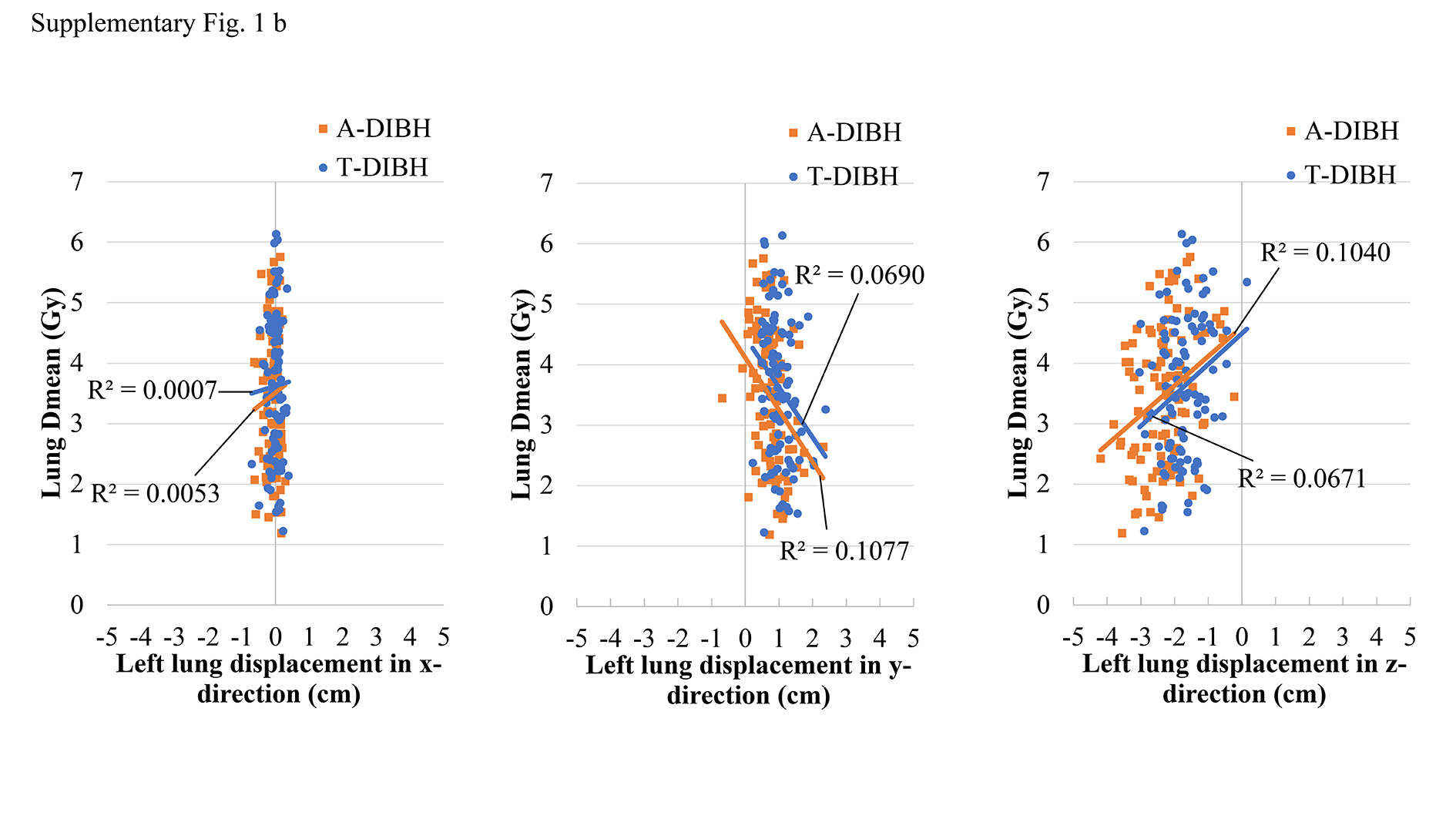

Supplement: Supplementary file 2 — Supporting Material [file ACM2-24-e13888-s006.tif]

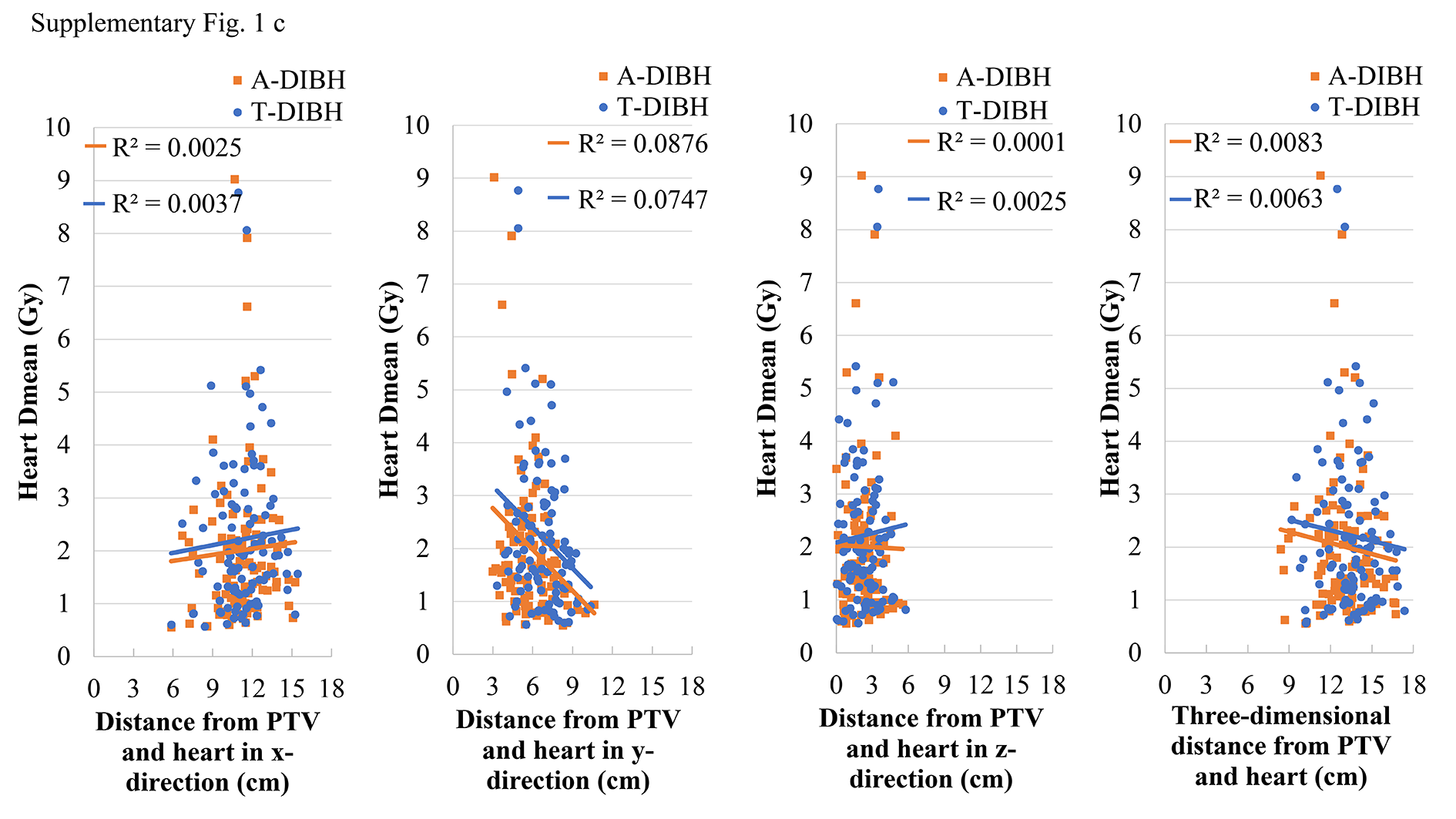

Supplement: Supplementary file 3 — Supporting Material [file ACM2-24-e13888-s008.tif]

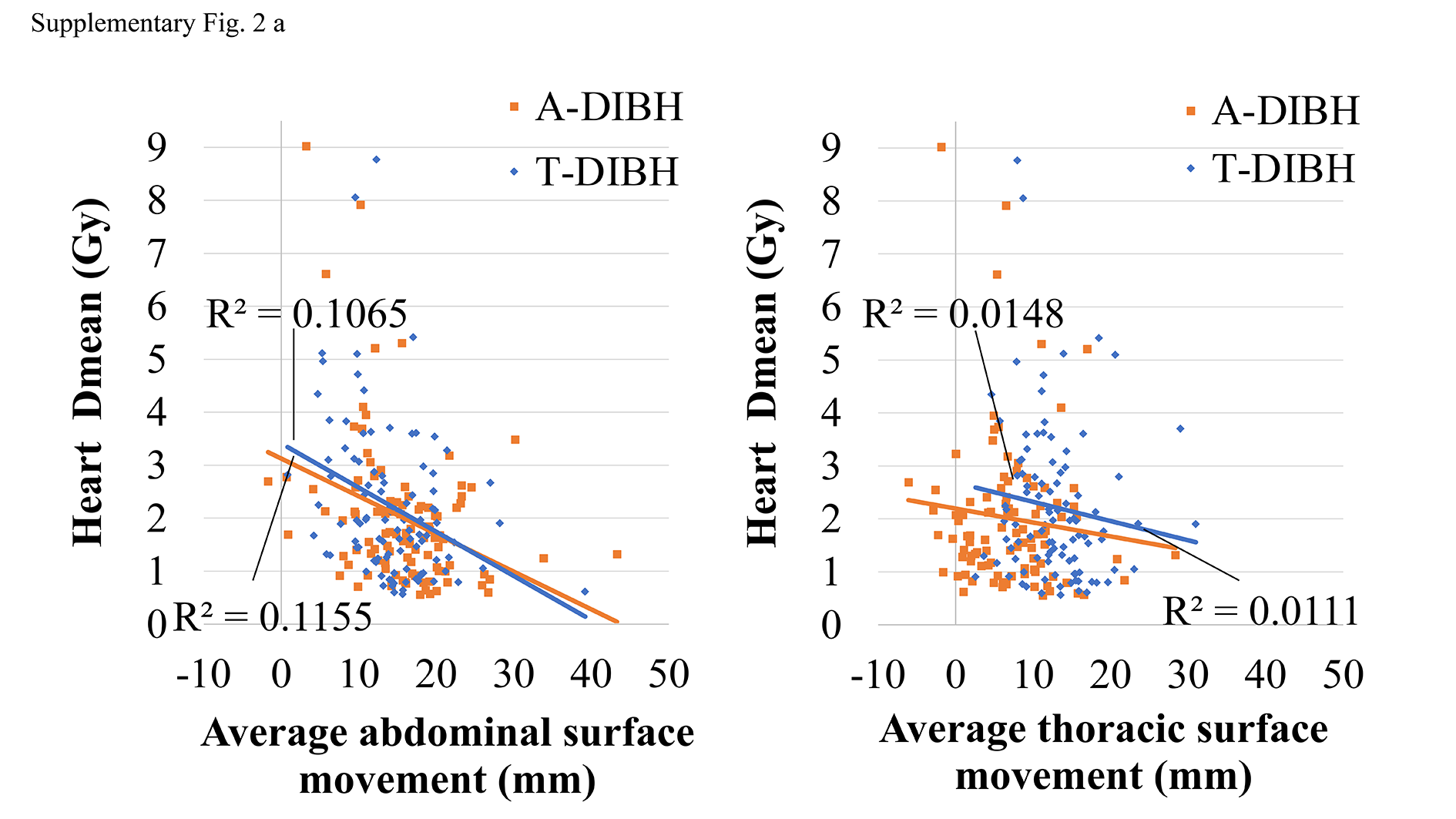

Supplement: Supplementary file 4 — Supporting Material [file ACM2-24-e13888-s003.tif]

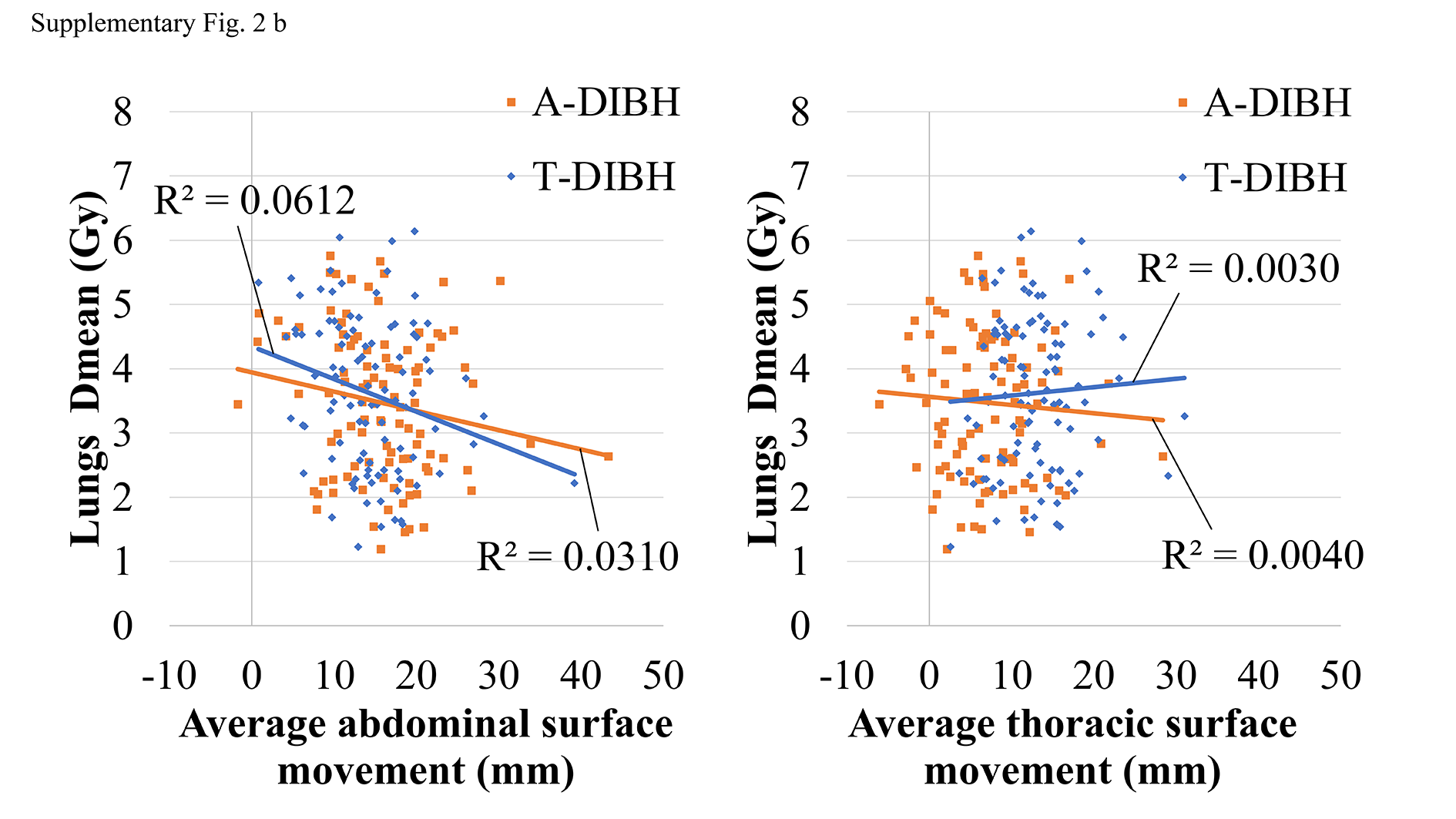

Supplement: Supplementary file 5 — Supporting Material [file ACM2-24-e13888-s001.tif]

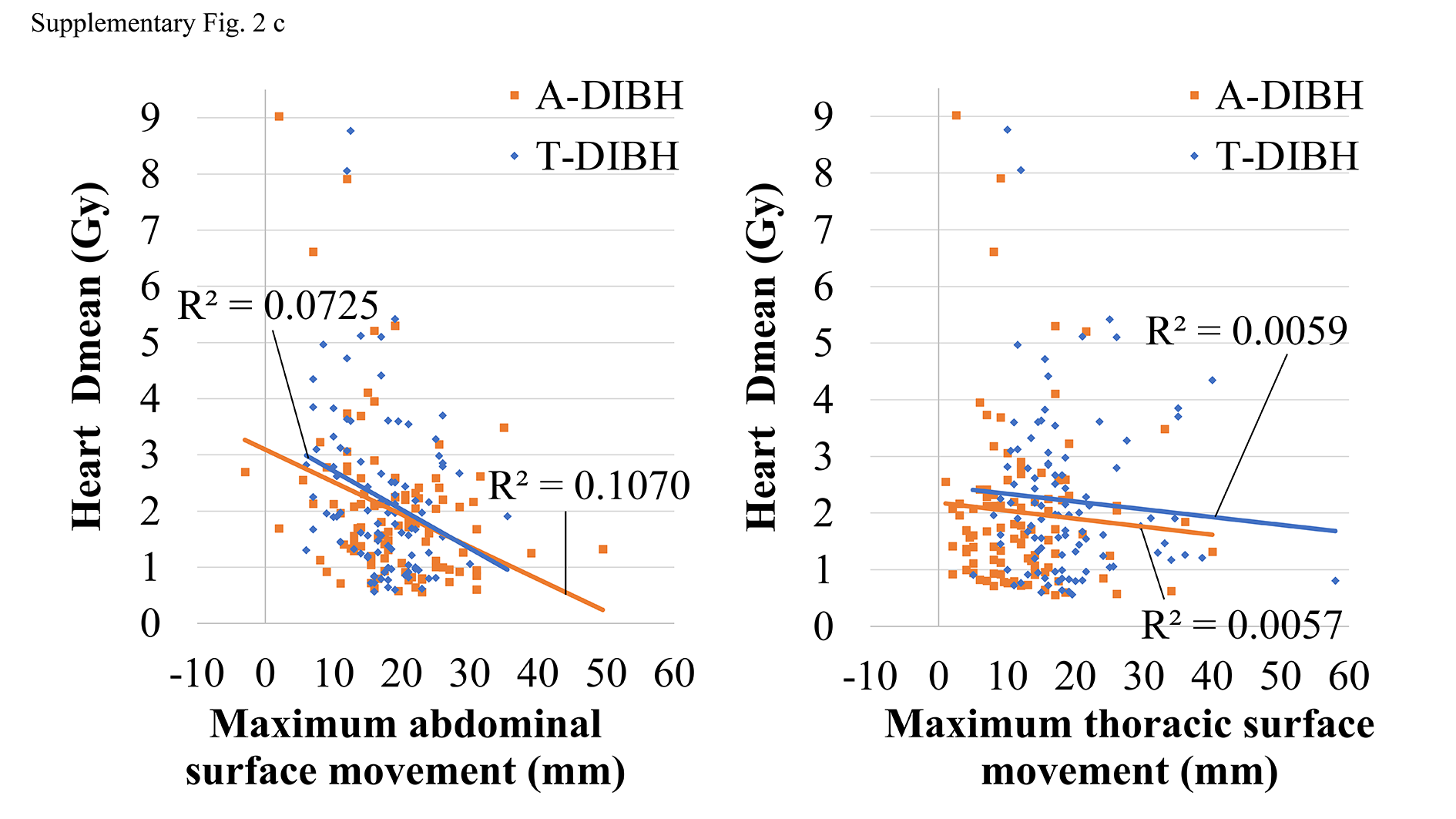

Supplement: Supplementary file 6 — Supporting Material [file ACM2-24-e13888-s004.tif]

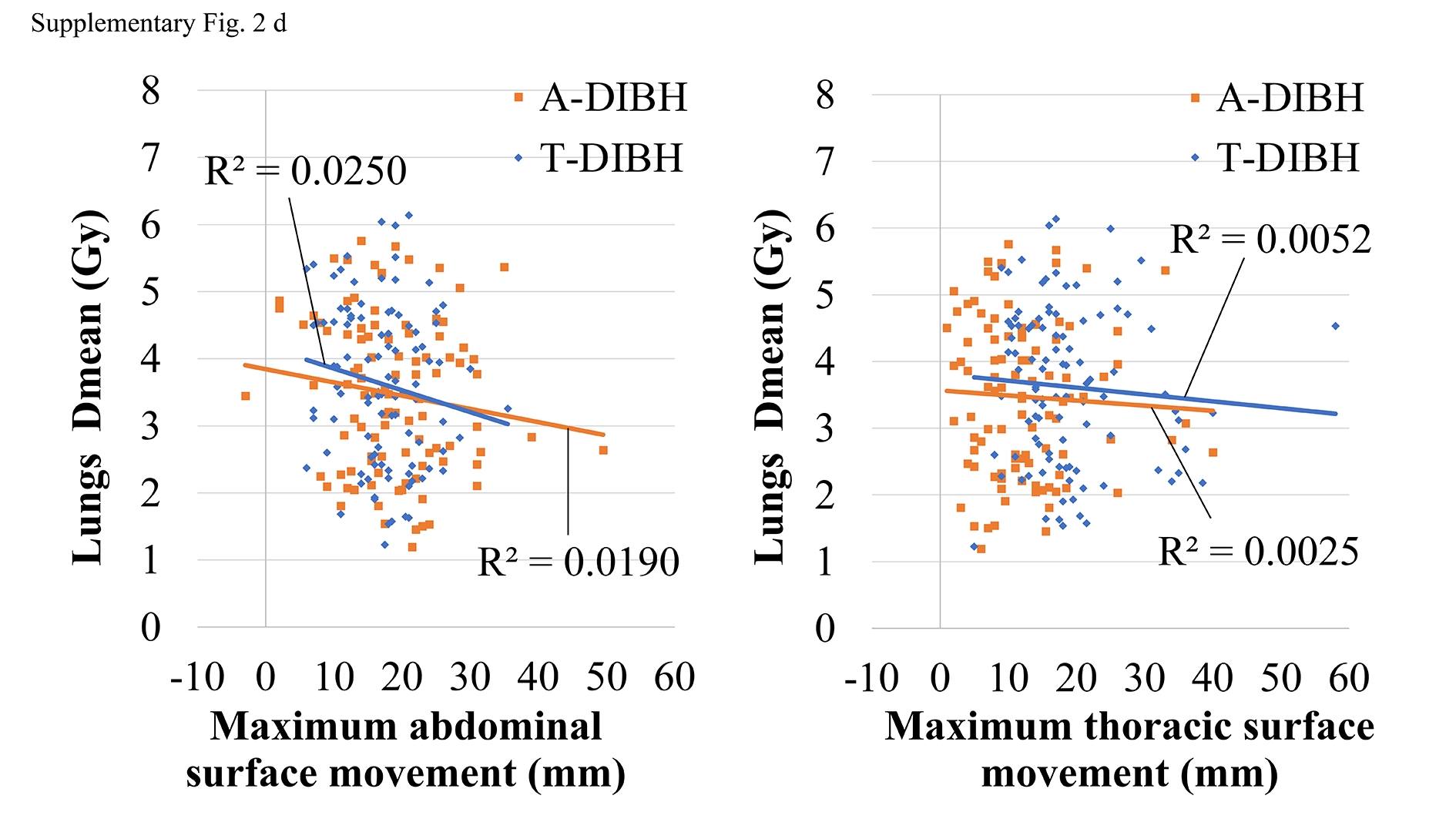

Supplement: Supplementary file 7 — Supporting Material [file ACM2-24-e13888-s007.tif]
