# Supplementary material for: Dose and organ displacement comparisons with breast conservative radiotherapy using abdominal and thoracic deep‐inspiration breath‐holds: A comparative dosimetric study
Source: J Appl Clin Med Phys. 2023 Jan 7;24(4):e13888. doi: 10.1002/acm2.13888 (PMC10113706; doi:10.1002/acm2.13888)
Supplement: Supplementary file 8 — Supporting Material [file ACM2-24-e13888-s005.pdf]

Supplementary Table 1: Comparison between non-modified MLC and modified MLC on A-DIBH and T-DIBH for each index

Parameters		A-DIBH	A-DIBH (Modify)	T-DIBH (Modify)	*p-value* [A-DIBH vs A-DIBH (Modify)]	*p-value* A-DIBH [(Modify) vs T-DIBH (Modify)]
PTV						
	D98% (Gy)	22.91 (18.20–28.19)	22.49 (18.19–28.17)	21.13 (18.04–25.62)	**0.001 ****	0.421
	D95% (Gy)	29.49 (22.92–34.17)	29.46 (22.89–34.13)	31.30 (24.97–35.05)	**0.001 ****	0.247
	D50% (Gy)	39.40 (38.46–40.04)	39.33 (38.37–40.04)	39.39 (38.53–40.1)	**0.001 ****	0.179
	D2% (Gy)	41.38 (40.32–41.69)	41.33 (40.25–41.69)	41.41 (40.35–41.88)	0.172	0.083
	HI	0.46 (0.34–0.60)	0.46 (0.34–0.60)	1.05 (0.71–2.05)	1.000	0.155
Heart						
	Mean (Gy)	1.78 (0.90–5.30)	1.10 (0.81–1.66)	1.87 (0.78–5.42)	**0.000****	0.370
	V30 Gy (%)	0.98 (0.00–9.15)	0.00 (0.00–0.30)	0.00 (0.00–0.78)	**0.000 ****	0.933
	V20 Gy (%)	1.86 (0.00–11.07)	0.09 (0.00–0.78)	0.07 (0.00–1.69)	**0.000 ****	0.711
	V10 Gy (%)	3.06 (0.02–13.33)	0.61 (0.02–1.67)	0.56 (0.02–3.03)	**0.000 ****	0.911
	V5 Gy (%)	5.04 (0.38–16.11)	2.00 (0.38–3.92)	1.98 (0.40–6.04)	**0.000 ****	0.955
Lungs						
	Mean (Gy)	4.01 (1.54–5.68)	3.90 (1.53–5.32)	3.91 (1.56–5.28)	**0.000 ****	0.247
	V30 Gy (%)	6.56 (1.44–11.65)	5.88 (1.43–10.09)	6.57 (1.43–10.42)	**0.000 ****	0.117
	V20 Gy (%)	8.62 (2.21–13.86)	8.32 (2.18–12.42)	8.73 (2.29–12.83)	**0.000 ****	0.156

	V10 Gy (%)	10.89 (3.25–15.60)	10.54 (3.22–14.81)	10.72 (3.42–14.84)	**0.000 ****	0.247
	V5 Gy (%)	15.24 (5.78–21.25)	14.95 (5.75–20.75)	15.37 (6.53–20.35)	**0.000 ****	0.332
LAD	Maximum (Gy)	37.21 (8.22–40.32)	31.68 (8.22–37.49)	31.53 (13.06–38.27)	**0.000 ****	0.467
	Mean (Gy)	18.91 (0.00–59.81)	8.38 (2.65–16.04)	8.36 (3.65–24.13)	**0.000 ****	0.156
	V15 Gy (%)	29.26 (0.00–67.91)	18.91 (0.00–56.79)	21.7 (0–72.77)	**0.000 ****	0.099
LV	Maximum (Gy)	38.10 (17.14–39.90)	26.11 (17.14–37.38)	25.54 (12.31–37.22)	**0.030 ***	0.478
	Mean (Gy)	3.31 (1.16–10.13)	1.67 (1.16–3.09)	1.54 (1.12–3.18)	**0.000 ****	0.506
	V15 Gy (%)	6.13 (0.01–25.57)	0.43 (0.01–2.99)	0.31 (0–3.27)	**0.000 ****	0.081

*** P<0.01, * P<0.05*

Data are presented as median (range).

MLC, multi-leaf collimator; A-DIBH, abdominal deep-inspiration breath-hold; T-DIBH, thoracic deep-inspiration breath-hold; HI, homogeneity index; PTV, planning target volume; Modify, the multi-leaf collimator was modified to shield the heart.
