# Supplementary material for: Dose and organ displacement comparisons with breast conservative radiotherapy using abdominal and thoracic deep‐inspiration breath‐holds: A comparative dosimetric study
Source: J Appl Clin Med Phys. 2023 Jan 7;24(4):e13888. doi: 10.1002/acm2.13888 (PMC10113706; doi:10.1002/acm2.13888)
Supplement: Supplementary file 9 — Supporting Material [file ACM2-24-e13888-s002.pdf]

Supplementary Table 2: Comparison between AAA and AXB on A-DIBH and T-DIBH for each index

Parameters		A-DIBH (AAA)	A-DIBH (AXB)	T-DIBH (AXB)	*p-value* [A-DIBH (AAA) vs A-DIBH (AXB)]	*p-value* [A-DIBH (AXB) vs T-DIBH (AXB)]
PTV						
	D98% (Gy)	22.91 (18.20–28.19)	25.97 (19.67–31.16)	23.22 (19.68–29.28)	**0.000 ****	0.204
	D95% (Gy)	29.49 (22.92–34.17)	32.53 (26.01–35.60)	31.30 (24.97–35.05)	**0.000 ****	0.179
	D50% (Gy)	39.40 (38.46–40.04)	39.19 (38.33–39.98)	39.29 (38.33–39.98)	**0.003 ****	**0.029 ***
	D2% (Gy)	41.38 (40.32–41.69)	41.57 (40.75–42.30)	41.57 (40.75–40.11)	**0.001 ****	0.076
	HI	0.46 (0.34–0.60)	0.40 (0.27–0.56)	0.48 (0.31–0.55)	**0.000 ****	0.224
Heart						
	Mean (Gy)	1.78 (0.90–5.30)	1.78 (0.84–5.26)	1.87 (0.78–5.42)	**0.001 ****	0.067
	V30 Gy (%)	0.98 (0.00–9.15)	0.93 (0.00–8.94)	1.50 (0.00–9.42)	**0.001 ****	**0.044 ***
	V20 Gy (%)	1.86 (0.00–11.07)	1.82 (0.00–11.11)	2.44 (0.00–11.55)	**0.001 ****	**0.030 ***
	V10 Gy (%)	3.06 (0.02–13.33)	3.09 (0.02–13.37)	3.38 (0.04–13.86)	0.099	**0.023 ***
	V5 Gy (%)	5.04 (0.38–16.11)	4.60 (0.29–15.74)	4.56 (0.28–16.45)	**0.000 ****	**0.040 ***
Lungs						
	Mean (Gy)	4.01 (1.54–5.68)	3.97 (3.77–16.15)	4.10 (1.55–5.99)	**0.000 ****	0.108
	V30 Gy (%)	6.56 (1.44–11.65)	5.87 (1.07–11.13)	6.70 (1.13–12.35)	**0.000 ****	**0.035 ***
	V20 Gy (%)	8.62 (2.21–13.86)	8.58 (2.22–13.80)	9.25 (2.33–14.66)	**0.000 ****	0.059

	V10 Gy (%)	10.89 (3.25–15.60)	11.67 (3.77–16.15)	11.78 (3.99–16.53)	**0.002 ****	0.179
	V5 Gy (%)	15.24 (5.78–21.25)	15.82 (6.29–21.19)	15.37 (6.53–20.35)	**0.000 ****	0.167
LAD	Maximum (Gy)	37.21 (8.22–40.32)	37.40 (7.94–40.14)	37.82 (13.05–39.72)	0.533	0.455
	Mean (Gy)	18.91 (0.00–59.81)	11.38 (2.21–24.10)	12.25 (3.41–29.13)	**0.000 ****	0.135
	V15 Gy (%)	29.26 (0.00–67.91)	29.43 (0.00–67.6)	32.80 (0.00–82.84)	0.777	0.159
LV	Maximum (Gy)	38.1 (17.14–39.90)	37.76 (16.65–39.60)	37.99 (13.20–40.08)	**0.013 ***	0.550
	Mean (Gy)	3.31 (1.16–10.13)	3.22 (1.06–9.99)	3.89 (1.02–11.24)	**0.000 ****	**0.020 ***
	V15 Gy (%)	6.13 (0.01–25.57)	6.14 (0.01–25.61)	8.33 (0.00–28.93)	0.913	**0.019 ***

*** P<0.01, * P<0.05*

Data are presented as median (range).

AAA, analytical anisotropic algorithm; AXB, Acuros XB algorithm; A-DIBH, abdominal deep-inspiration breath-hold; T-DIBH, thoracic deep-inspiration breath-hold; HI, homogeneity index; PTV, planning target volume.
